# Taohong Siwu Decoction Regulates MSC‐Mediated H‐Type Angiogenesis to Accelerate Bone Fracture Healing Through VHL/HIF‐1α Ubiquitination

**DOI:** 10.1155/mi/6551954

**Published:** 2026-02-16

**Authors:** Wangyang Li, Zebing Ma, Peng He, Wuji Xu, Xiaolan Liu, Jinlong Yao, Qiyao Wu, Pinglan Zou, Tiao Li

**Affiliations:** ^1^ Department of Orthopedics and Traumatology, The Second Affiliated Hospital of Hunan University of Chinese Medicine, Changsha, China; ^2^ Chinese Medicine College, Changsha Medical University, Changsha, China, csmu.edu.cn; ^3^ College of Pharmacy, Hunan University of Chinese Medicine, Changsha, China, hnctcm.edu.cn; ^4^ Department of Respiratory Medicine, National Key Clinical Specialty, Branch of National Clinical Research Center for Respiratory Disease, Xiangya Hospital, Central South University, Changsha, China, csu.edu.cn

**Keywords:** bone fracture healing, HIF-1α ubiquitination, H-type blood vessels, MSCs, Taohong Siwu decoction

## Abstract

**Background:**

Bone fracture healing is a multifaceted process that involves different stages and intercellular interactions. In this study, we aimed to investigate the effect of Taohong Siwu decoction (TSD) on bone fracture healing and the underlying mechanisms.

**Methods:**

First, a mouse model of femur fracture was constructed, and TSD intervention was administered for durations of 7, 14, and 21 days. Following this, immunofluorescence (IF) was employed to evaluate the expression of CD90 (a marker for mesenchymal stem cells [MSCs]), endomucin (Emcn), and CD31. We also treated MSCs with normal serum and 10% TSD‐containing serum to investigate the effects of TSD. Molecular docking was applied to verify the binding of active compounds in TSD to pVon Hippel–Lindau (VHL). Additionally, MSCs were treated with paeoniflorin and 2‐methoxyestradiol (2‐ME2) to explore the effects of paeoniflorin. Subsequently, mouse aortic endothelial cells were extracted and identified. Furthermore, normally cultured MSCs were cocultured with endothelial cells. MSCs were exposed to control serum, 10% TSD‐containing serum, and a combination of 10% TSD‐containing serum with 2‐ME2. Finally, we administered a combination of 2‐ME2 over 21 days to evaluate its effects on the fractured mice.

**Results:**

TSD significantly influenced H‐type angiogenesis during the healing process of fractured mice. Compared to the sham group, the model group exhibited lower levels of Emcn, CD90, hypoxia‐inducible factor‐1 alpha (HIF‐1α), and vascular endothelial growth factor (VEGF), while there was an increase in pVHL expression. After 7, 14, and 21 days of TSD intervention, the levels of Emcn, CD90, HIF‐1α, VEGF, and pVHL gradually increased, whereas HIF‐1α expression decreased. In vitro experiments revealed that TSD enhanced the proliferation and migration of MSCs while inhibiting the ubiquitination of pVHL/HIF‐1α. Moreover, ferulic acid, amygdalin, hydroxysafflor yellow A, and paeoniflorin demonstrated a strong affinity for binding with pVHL. Notably, paeoniflorin promoted the proliferation and migration of MSCs through the pVHL/HIF‐1α pathway to promote angiogenesis. Furthermore, TSD was found to enhance endothelial angiogenesis in MSCs. In summary, TSD affects H‐type angiogenesis and MSCs homing during the healing process of fractured mice through the HIF‐1α axis.

**Conclusions:**

TSD regulated MSC‐mediated H‐type angiogenesis to accelerate fracture healing through VHL/HIF‐1α ubiquitination.

## 1. Introduction

Bone fractures are the most prevalent type of musculoskeletal injury worldwide [1]. With the development of an aging population, bone fractures have emerged as a significant public health issue due to their high incidence, associated disability rates, and mortality [2]. The process of healing bone fractures is complex and dynamic that aims to restore the damaged bone to its original condition and cellular structure [3]. The fracture healing process consists of four distinct but overlapping stages: hematoma formation, cartilage callus development, bone callus development, and fracture callus remodeling [4]. Although the goal of fracture repair is to restore the damaged bone organ to its previous cellular composition, structure, and biomechanical function, ~10% of fractures fail to heal properly [5]. Consequently, there is an urgent need to explore new treatments that could enhance the healing of fractures.

Taohong Siwu decoction (TSD) is a traditional Chinese medicine formula consisting of several key ingredients: *Persicae Semen* (Taoren), *Carthami Flos* (Honghua), *Angelicae Sinensis Radix* (Danggui), *Rehmanniae Radix Praeparata* (Shudihuang), *Chuanxiong Rhizoma* (Chuanxiong), and *Paeoniae Radix Alba* (Baishao) [6]. This classic prescription is intended to promote blood circulation and alleviate blood stasis, effectively nourishing the blood and restoring normal bodily functions [7]. Our previous studies have demonstrated that TSD promotes angiogenesis by modulating the Von Hippel–Lindau (VHL)/hypoxia‐inducible factor‐1 alpha (HIF‐1α)/vascular endothelial growth factor (VEGF) pathway [8]. Additionally, TSD may accelerate the healing of bone fractures and enhance the homing of mesenchymal stem cells (MSCs) to the fracture site [9]. Consequently, this study aims to explore further the novel mechanisms by which TSD promotes bone fracture healing.

Bone formation and remodeling are processes that depend on both vascularization and osteogenesis [10]. During these processes, new microvessels and homing MSCs supply nutrients and osteoblasts, which are essential for bone tissue regeneration.

These elements are critical for initiating the regeneration of bone tissue [11–13]. Within the bone, a specialized type of capillary blood vessel, known as the H‐type, exists. This vessel is characterized by high expression levels of PECAM‐1 (CD31) and endomucin (Emcn) and is capable of inducing both angiogenesis and bone formation. Enhancing H‐type angiogenesis could significantly promote bone mass recovery and play a critical role in fracture healing [14]. Nonetheless, it remains unclear whether TSD affects H‐type angiogenesis and promotes fracture healing by regulating the homing of MSCs, an area that warrants further exploration.

Fracture healing is a complex biological process that involves the interplay of various cellular and molecular signaling pathways [15]. VHL is a key E3 ubiquitin ligase in the HIF‐1α ubiquitination degradation pathway. It specifically binds to hydroxylated HIF‐1α, mediating its proteasomal degradation [16]. When VHL expression is inhibited, it disrupts the balance between HIF‐1α synthesis and degradation, resulting in the intracellular accumulation of HIF‐1α, which subsequently promotes angiogenesis [17]. In this study, we aimed to investigate whether TSD (the specific agent under investigation) enhances fracture healing by modulating the VHL/HIF‐1α ubiquitination pathway, thereby influencing MSC‐mediated H‐type angiogenesis. We employed a mouse femur fracture model, along with molecular docking and in vitro experiments, to identify potential targets and molecular mechanisms through which TSD may facilitate fracture healing. Our findings are intended to serve as valuable references for future research in this field.

## 2. Materials and Methods

### 2.1. Construction of the Mouse Femur Fracture Model

Eight‐week‐old male BALB/c mice were procured from Hunan SJA Laboratory Animal Co., Ltd. The mice were housed in a temperature‐controlled animal facility with a 12‐h light/12‐h dark cycle and were allowed 1 week to acclimate to their environment on standard rodent chow. The mice were randomly assigned to one of five groups: sham, model, TSD‐7d, TSD‐14d, and TSD‐21d, with six mice in each group. The mice were anesthetized via intraperitoneal injection of 1% sodium pentobarbital (0.1 mL/100 g). After disinfecting and draping, an incision was made on the medial part of the patella of the left lower limb, ~0.5 cm in length. The patella was then carefully dislocated laterally to expose the intercondylar fossa of the femur. A 26‐gauge injection needle was inserted into the intercondylar fossa to expand the medulla oblongata, after which the needle was removed. Subsequently, a Kirschner needle (2 mm in diameter) was inserted retrogradely through the skin from the greater trochanter. The distal end of the Kirschner needle was positioned beneath the cortex of the femur, while the proximal end was bent and cut just outside the greater trochanter. The skin was then sutured closed after the bone was disinfected with 75% alcohol, and an intraperitoneal injection of 80,000 units of penicillin sodium was administered [18, 19]. On the first postoperative day, X‐rays were taken to confirm the success of the fracture modeling. After establishing the fracture model, the mice in the TSD‐7d group underwent TSD (27 g/kg) intervention for 7 days. Those in the TSD‐14d group underwent TSD (27 g/kg) for 14 days, whereas the TSD‐21d group received the same intervention for 21 days [20]. The decoction was prepared according to the Standard for Management of TCM Decocting Room in Medical Institutions and Technical Requirements for Quality Control and Standard Formulation of TCM Granules [21]. The medicinal materials used in TSD were purchased from the certified traditional Chinese medicinal materials supplier Hunan Sanxiang Traditional Chinese Medicine Slices Co., Ltd., and have passed strict quality inspections to ensure compliance with national pharmacopeia standards. Additional groups included the model group, TSD group, 2‐methoxyestradiol (2‐ME2) group, and TSD + 2‐ME2 group, with each consisting of six mice. Mice in the TSD group underwent TSD (27 g/kg) intervention for 21 days after fracture modeling [20]. The TSD group received the TSD (27 g/kg) intervention for 21 days following fracture modeling. Mice in the 2‐ME2 group were treated with 2‐ME2 (100 mg/kg, S1233, Selleck) for 21 days after modeling. The TSD + 2‐ME2 group was subjected to both TSD (27 g/kg) and 2‐ME2 (100 mg/kg) treatments for 21 days post‐fracture modeling [22, 23]. Following the construction of the mouse femur fracture model, MSC cells were isolated from bone marrow [24]. The MSCs were cultured in a cell‐specific complete medium (AW‐MC025, Abiowell). All experimental procedures and animal handling were conducted with the approval of the Animal Care and Use Committee of the Second Affiliated Hospital of Hunan University of Chinese Medicine (Number HNUCM21‐2310‐02), in compliance with the National Institutes of Health Guide for the Care and Use of Laboratory Animals. Furthermore, studies involving laboratory animals adhered to the ARRIVE guidelines.

### 2.2. Preparation of TSD‐Containing Serum

The dosage of TSD administered via gavage was 27 g/kg. Male mice were acclimatized for 1 week, after which they were fed TSD via gavage twice daily. The control group received an equal volume of saline via gavage for 7 days. Following the final gavage, the mice were anesthetized with 1% sodium pentobarbital (0.1 mL/100 g) 2 h later. Blood samples were then collected using blood collection tubes and stored at 4°C. The blood samples were initially filtered through a 0.22 μm pore‐sized filter to eliminate any potential particulate impurities. Subsequently, the samples were heat‐inactivated at 56°C to neutralize any potential pathogens. The processed samples were then immediately stored in an ultra‐low‐temperature freezer at −80°C for subsequent experimental use.

### 2.3. Isolation and Identification of Mouse Aortic Endothelial Cells

Under sterile conditions, the thoracic and abdominal aorta of mice were isolated. Tweezers were used to carefully strip away the fat and connective tissue surrounding the aorta. The aorta was then opened under a dissecting microscope to create a flat vascular slice, which was subsequently placed in Type I collagenase (1 mg/mL, SCR103, Sigma) and digested at 37°C for 30 min. After digestion, the process was terminated by adding complete medium containing 10% FBS. The vascular slice was gently and repeatedly pipetted 50–100 times to flush the endothelial cells from the intima. The resulting cell suspension was centrifuged at 1000 rpm for 5 min, and the supernatant was carefully discarded. The harvested endothelial cells were then cultured in a primary endothelial cell‐specific complete medium (AW‐MC013, Abiowell). After 24 h, a half‐volume medium change was performed to remove non‐adherent cells and debris, with subsequent medium changes occurring every 3–4 days to support ongoing cell culture. When the cell density reached ~80% of the culture flask area, the cells were passaged. After 8–9 days of culture, a high‐magnification microscope was used to observe the morphology of the isolated cells. Flow cytometry and immunofluorescence (IF) were used to identify the expression of CD31, a marker for vascular endothelial cells, which enabled the successful culture of primary mouse aortic endothelial cells in vitro. The isolated endothelial cells were maintained in standard medium and used at 2–4 passages [25].

### 2.4. Cell Culture and Treatment

MSCs were cultured in cell‐specific complete medium (AW‐MC025, Abiowell), and mouse aortic endothelial cells were cultured in endothelial cell‐specific complete medium (AW‐MC013, Abiowell) at 37°C, with 5% CO_2_, in a saturated humidity incubator. Logarithmically grown MSCs were treated in the following groups: control group (MSCs cultured normally), control serum group (MSCs treated with normal serum for 24 h), and TSD serum group (MSCs treated with 10% TSD‐containing serum for 24 h). MSCs transfected with oe‐VHL were incubated with 20 μmol/L MG132 (HIF‐1α inhibitor, HY‐13259, MCE) for 2 h before simulation with 10% TSD‐containing serum [26]. The groups were designated as oe‐NC, oe‐VHL, and oe‐VHL + MG132. Additionally, MSCs were treated with different concentrations of paeoniflorin (HY‐N0293, MCE) [27] and divided into control, DMSO, 20, 40, 60, 80, 100, and 120 μM paeoniflorin groups. After identifying the optimal treatment concentration, 2‐ME2 (HIF‐1α inhibitor) was applied, resulting in the following groups: control, DMSO, 80 μM paeoniflorin, and 80 μM paeoniflorin + 2‐ME2. Additionally, MSCs were divided into control, DMSO, paeoniflorin, paeoniflorin+oe‐NC, and paeoniflorin+oe‐VHL groups. Moreover, MSCs were cocultured with endothelial cells to explore how MSCs influence the angiogenesis process through direct interaction with endothelial cells. The groups for this experiment included control group (normal cultured MSCs cocultured with endothelial cells for 24 h), control serum group (normal serum‐treated MSCs cocultured with endothelial cells for 24 h), TSD serum group (10% TSD‐containing serum‐treated MSCs cocultured with endothelial cells for 24 h), and TSD serum + 2‐ME2 group (MSCs treated with 10% TSD‐containing serum and 10 μM 2‐ME2 cocultured with endothelial cells for 24 h) [22, 23]. MSCs in the control, DMSO, 80 μM paeoniflorin, and 80 μM paeoniflorin + 2‐ME2 groups were cocultured with endothelial cells. Furthermore, MSCs in the control, DMSO, paeoniflorin, paeoniflorin+oe‐NC, and paeoniflorin+oe‐VHL groups were cocultured with endothelial cells. The cells were cocultured in a 1:1 ratio with MSCs in the upper chamber and endothelial cells in the lower chamber. Transfection was performed using Lip 2000 (11668019, Invitrogen).

### 2.5. Micro‐CT Scanning

After the experimental intervention, the right femoral shaft of the mouse was removed along with excess muscle tissue. It was then fixed with 4% paraformaldehyde and placed in a micro‐CT test tube in a longitudinal orientation. The region of interest was defined as the area extending 5 mm above and below the center of the fracture site. Following Gaussian filtering, a maximum grayscale threshold of 30% was established to distinguish between mineralized and nonmineralized tissues. Through system software analysis, the tissue volume (TV), bone volume (BV), BV fraction (BV/TV[%]), and bone mineral density (BMD) of the fracture site of the mouse femur were determined.

### 2.6. Hematoxylin–Eosin (H&E) Staining

To prepare the mouse femur for histological analysis, an H&E staining kit (AWI0020, Abiowell) was utilized. The sections were baked at 60°C for 12 h and then dewaxed in water. They were subsequently immersed in xylene (W990018, Shanghai Pharmaceuticals Holding Co., Ltd) for 20 min for three cycles. Subsequently, the sections were sequentially immersed in 100%, 100% again, 95%, 85%, and 75% ethanol, with each immersion lasting 5 min. Next, the sections were stained with hematoxylin for 1–10 min and then blued in PBS. Eosin staining was then applied for 1–5 min. After the staining process, the sections were dehydrated in graded alcohol (95%–100%) for 5 min or dried directly by baking. Afterwards, the sections were placed in xylene for 10 min in two changes, followed by sealing with neutral gum (G8590, Solarbio). The sections were then observed under a microscope.

### 2.7. Safranin O‐Fast Green Staining

Safranin O‐fast green staining kit (AWI0240, Abiowell) was employed to evaluate changes in bone mass distribution in mouse femurs. The sections were initially baked, deparaffinized, and immersed in xylene for 10 min twice. The sections were sequentially immersed in 100%, 100%, 95%, 85%, and 75% ethanol, with each immersion lasting 5 min. The solid green staining solution was applied for about 5 min, after which the sections were washed twice with 1% acetic acid. Saffron dye was then applied for about 30 s. Following drying with a hair dryer, the sections were treated with xylene for two cycles of 10 min each. Finally, they were sealed with neutral gum and examined under a microscope.

### 2.8. Flow Cytometry

Flow cytometry was conducted to measure CD31 expression on the surface of endothelial cells. In 1.5 mL EP tubes, 1 × 10^6^ cells/100 μL was taken and incubated with CD31 antibody at room temperature, shielded from light, for 30 min. The cells were then washed with 1 mL of PBS, centrifuged at 350 × *g* for 5 min, and the supernatant was discarded. Following a second wash with 1 mL of PBS and centrifugation at 350 × *g* for 5 min, the cells were resuspended in 350 μL of PBS and analyzed using flow cytometry (A00‐1‐1102, Beckman).

### 2.9. IF

IF was used to evaluate the expression of CD90, Emcn, and CD31 in mouse femur tissues. The tissue sections were baked and deparaffinized in water. Subsequently, the sections were incubated in urea (ST1731, Beyotime) at 37°C for 30 min and then washed with 0.01 M PBS (pH 7.2–7.6) for 3 min. Following this, the sections were incubated in trypsin (25200056, Gibco) at 37°C for 30 min and then rinsed with 0.01 M PBS (pH 7.2–7.6) for 3 min. The sections were immersed in a sodium borohydride solution (215511, Sigma) for 30 min to block endogenous oxidants. Subsequently, sections were blocked in a 5% BSA solution (MB4219‐3, Dalian Meilun Biotech Co., Ltd) for 60 min. The sections were incubated with CD90 (27178‐1‐AP, 1:100, Proteintech) or CD31 (AWA00516, 1:200, Abiowell) overnight at 4°C and Dual‐label Multiplex IF Assay Kit (for paraffin‐embedded sections) (AWI0692, Abiowell) at 37°C for 31 min. The TYP‐520 fluorescent dye was then incubated at 37°C for 5–10 min, protected from light, followed by rinsing in PBS for 5 min. For the detection of Emcn and CD31 expressions, an appropriate amount of antibody eluate, prewarmed at 37°C until completely dissolved, was added dropwise to cover the samples. The samples were then left at 37°C for 10 min to discard the eluate, and the process was repeated once. Sections were then incubated with urea at 37°C for 30 min and washed with 0.01 M PBS (pH 7.2–7.6) for 3 min. Subsequently, sections were incubated in trypsin at 37°C for 30 min and then washed with 0.01 M PBS (pH 7.2–7.6) for 3 min. Endogenous oxidants were blocked, and sections were then blocked in a 10% normal serum/5% BSA solution for 60 min. Sections were incubated with Emcn (AWA58020, 1:100, Abiowell) overnight at 4°C and Dual‐label Multiplex IF Assay Kit (for paraffin‐embedded sections) (AWI0692, Abiowell) at 37°C for 31 min. TYP‐570 fluorescent dye was incubated at 37°C for 5–10 min and protected from light. Finally, the nuclei were stained with DAPI (AWC0293a, Abiowell) at 37°C for 10–20 min. Buffered glycerol was applied to seal sections, and they were stored away from light or observed under a fluorescence microscope.

Additionally, IF was used to detect the expression of CD31 in mouse aortic endothelial cells. The cover slips were removed and washed with PBS two to three times. The cover slips were then fixed with 4% paraformaldehyde for 30 min and rinsed with PBS three times, each for 5 min. A 0.3% Triton X‐100 solution (X100, Sigma) was added for permeabilization at 37°C for 30 min, followed by three rinses with PBS for 3 min each. A 5% BSA solution was applied at 37°C for 60 min, and then the coverslips were rinsed with PBS three times for 3 min each. An appropriately diluted primary antibody against CD31 (AWA03811, 1:50, Abiowell) was added and incubated overnight at 4°C. Afterward, the cover slips were rinsed three times with PBS for 5 min each. Then, 50–100 μL of Goat anti‐Mouse IgG (H + L) Secondary Antibody, Alexa Fluor 488 (AWS0003, Abiowell), was added and incubated at 37°C for 90 min, followed by three rinses with PBS for 5 min each. The nuclei were stained with a DAPI working solution at 37°C for 10 min and then rinsed with PBS three times for 5 min each. Buffered glycerol was applied to seal cover slips. The samples were stored in the dark or observed under a fluorescence microscope.

### 2.10. Quantitative Real‐Time PCR (qRT‐PCR)

The expression of Osterix, Runx2, MMP‐9, PDGF‐BB, and SLIT3 was measured by qRT‐PCR. First, total RNA was extracted with the Trizol total RNA extraction kit (15596026, Thermo), and concentration and purity were determined. Next, the mRNA was reverse‐transcribed into complementary deoxyribonucleic acid (cDNA) using the mRNA reverse transcription kit (CW2569, CWBIO). The relative expression of genes was examined by applying Ultra SYBR Mixture (CW2601, CWBIO) on the ABI 7900 system. Gene expression was calculated using the 2^−ΔΔCt^ method, with β‐actin as the internal reference gene. The primers are shown below: Osterix‐F: TTGGATCTGAGTGGGAACAAGAG, Osterix‐R: TGAGCTTCTTCCTGGGTAGG; RUNX2‐F: ACTCCAAGACCCTAAGAAACCGAT, RUNX2‐R: TGGCTCCTCCCTTCTCAACCTC; MMP‐9‐F: GCCCTGGAACTCACACGACA, MMP‐9‐R: GTAGCCCACGTCGTCCACC; PDGF‐BB‐F: CCTCTCCCTGCAGTGAACTT, PDGF‐BB‐R: TCCTCCCTCGAGATGAGCTT; SLIT3‐F: AGTTGTCTGCCTTCCGACAG, SLIT3‐R: GCACTCGGAGGGATCTTAGC; and β‐actin‐F: ACATCCGTAAAGACCTCTATGCC, β‐actin‐R: TACTCCTGCTTGCTGATCCAC.

### 2.11. Cell Counting Kit 8 (CCK‐8) Assay

Cell viability was assessed using the CCK‐8 assay. The cells were digested, counted, and then seeded into 24‐well plates at a density of 5 × 10^3^ cells per well in 300 μL of medium. Three replicate wells were prepared for each group. After incubating the cells for the specified duration and allowing them to adhere to the well, 10 μL of CCK‐8 solution (NU679, DOJINDO) was added to each well and further incubated at 37°C with 5% CO_2_ for 4 h. Absorbance at 450 nm was measured using a microplate reader.

### 2.12. Transwell Assay

A Transwell assay was conducted to evaluate the cell migration ability. Cells were first resuspended in serum‐free medium at a concentration of 1 × 10^6^ cells/mL. Subsequently, 100 μL of cell suspension was added to the upper chamber of Transwell (33318035, Corning), while the lower chamber was filled with complete medium containing 10% FBS. The cells were then incubated for 48 h. After incubation, the culture medium was removed. Cells on the upper surface of the chamber were removed with a damp cotton swab. The remaining cells were fixed with 4% paraformaldehyde (P0099, Beyotime) for 10 min and stained with 0.5% crystal violet (C0121, Beyotime) for 5 min. Finally, cells on the outer surface of the upper chamber were observed and photographed under a microscope (Olympus, Japan).

### 2.13. Tube Formation Assay

The matrix gel (#356234, Biocoat) and a 96‐well plate were refrigerated at 4°C overnight. The matrix gel was then removed and placed in an icebox. Subsequently, 70 μL of matrix gel was added to each well of a precooled 96‐well plate to evenly coat wells, and the plate was allowed to stand at 4°C for 10 min. The plate was then placed in an incubator at 37°C for 30 min. Cells were trypsinized with 0.25% trypsin to create a cell suspension, and 10,000 cells were added to each well using a cell counter plate. The tube formation assay was conducted to assess the vascular ability of cells.

### 2.14. Western Blot

Western blot was conducted to assess pVHL, HIF‐1α, and VEGF levels and the nuclear expression of HIF‐1α. Total proteins were extracted from different sample groups using RIPA (AWB0136, Abiowell), separated by SDS‐PAGE, and transferred to nitrocellulose membranes. Following that, membranes were blocked with 5% skim milk and then incubated with primary antibodies overnight at 4°C. The primary antibodies used were pVHL (ab270968, 1:1000, Abcam), HIF‐1α (20960‐1‐AP, 1:6000, Proteintech), VEGF (ab32152, 1:3000, Abcam), PCNA (AWA43516, 1:1000, Abiowell), and β‐actin (AWA80002, 1:5000, Abiowell). Subsequently, the membranes were incubated with secondary antibodies, such as Goat anti‐Mouse (AWS0001, 1:5000, Abiowell), Goat anti‐Rabbit (AWS0002, 1:5000, Abiowell), or HRP‐conjugated Goat anti‐Rabbit IgG (SA00001‐2, 1:6000, Proteintech). Finally, the membranes were treated with ECL reagent (AWB0005, Abiowell) for 1 min and then analyzed using an imaging system. The expression levels of each protein were quantified using Quantity One 4.6.6.

### 2.15. HIF‐1α Ubiquitination Level Analysis

The target proteins were extracted and isolated from the control, control serum, and TSD serum groups. The ubiquitin antibody was added to the reaction buffer for in vitro incubation. The incubated products were then subjected to immunoprecipitation (IP) and western blot analysis.

### 2.16. Co‐IP

Co‐IP was conducted to confirm protein interactions between pVHL and HIF‐1α. Following IP using HIF‐1α antibody as the bait protein, pVHL and HIF‐1α levels were assessed by western blot. Initially, cells were harvested for protein extraction. Subsequently, equal amounts of protein extracts were subjected to IP in lysis buffer containing HIF‐1α antibody at 4°C overnight. Protein A/G agarose beads were then added to the IP mixture for 2 h, followed by three washes. Western blot analysis was performed using pVHL (ab270968, 1:1000, Abcam) and HIF‐1α (20960‐1‐AP, 1:6000, Proteintech) antibodies, followed by incubation with the corresponding secondary antibodies. Finally, the membranes were treated with ECL reagent (AWB0005, Abiowell) for 1 min and analyzed using an imaging system.

### 2.17. Molecular Docking

Based on our previous study [20], five compounds were detected in TSD, including ferulic acid, 5‐hydroxymethylfurfural, amygdalin, hydroxysafflor yellow A, and paeoniflorin. Molecular docking was applied to verify the active compounds in TSD binding to pVHL. In this study, the compounds and proteins were subjected to a docking study using VINA 1.1.2 software, which utilizes a semiempirical free ability field to predict binding energies of receptors and ligands. PYMOL was applied to analyze the compounds, which were able to bind stably into the cavity of the protein and interact with surrounding amino acids.

### 2.18. Drug Affinity Responsive Target Stability (DARTS) Assay

In accordance with previous research [28], DARTS assay was used to verify the binding of paeoniflorin with pVHL. The groups were divided as follows: control, pronase, pronase + 20 μM paeoniflorin, pronase + 40 μM paeoniflorin, pronase + 60 μM paeoniflorin, and pronase + 80 μM paeoniflorin. Additionally, DARTS assay was employed to verify the binding of 5‐hydroxymethylfurfural with pVHL. The groups were divided as follows: control, pronase, pronase + 20 μM 5‐hydroxymethylfurfural, pronase + 40 μM 5‐hydroxymethylfurfural, pronase + 60 μM 5‐hydroxymethylfurfural, and pronase + 80 μM 5‐hydroxymethylfurfural. pVHL levels were measured by western blot.

### 2.19. High‐Performance Liquid Chromatography (HPLC)

Compounds with the highest binding energy to pVHL in mouse blood were analyzed using liquid chromatograph‐–mass spectrometer (LC–MS). LC–MS analysis was conducted using an Agilent Technologies 1290 UHPLC system coupled with a combination of AB Sciex TripleTOF 6600 (Q‐TOF) and an Agilent Waters HSS T3 column (100 × 2.1 mm, 1.7 µm). Raw data were converted to mzXML format using ProteoWizard and processed utilizing a custom R software program developed in‐house. This program, built on XCMS, enabled peak detection, extraction, alignment, and integration. Metabolite annotations were performed using the in‐house MS2 database (BiotreeDB), with an annotation threshold set at 0.3.

### 2.20. Statistical Analysis

Statistical analysis was conducted using GraphPad Prism 8.0 software. The measurement data were expressed as mean ± standard deviation. Differences between two groups were assessed using the Student’s *t*‐test, whereas differences among multiple groups were evaluated using one‐way analysis of variance (ANOVA). A significance level of *p* < 0.05 was considered statistically significant.

## 3. Results

### 3.1. TSD Affected H‐Type Angiogenesis During Healing of Fractured Mice

First, a fracture model was constructed, and TSD intervention was performed for 7, 14, and 21 days. A micro‐CT scan was used to analyze the fracture ends of the mice, and a three‐dimensional map was established, as shown in Figure 1A. The TV/BV and BMD parameters were used to evaluate the volume of the callus at the fracture end and the degree of new bone mineralization. The BV/TV (%) represents the ratio of BV to total TV, which directly reflects the quantity of bone [29]. The BMD measures the amount of mineral content in the bone tissue of the region of interest [30]. As shown in Figure 1A, compared to the model group, the BV/TV and BMD significantly increased in the TSD‐7d, TSD‐14d, and TSD‐21d groups, with the TSD‐14d group showing the most significant increase. This indicated that TSD intervention promoted fracture healing and improved the quality of the healing process, with the changes being most evident in the TSD‐14d group. H&E staining demonstrated that, compared to the sham group, the model group exhibited some fibrous and cartilaginous callosities at the fracture ends of the femurs. In contrast, a substantial amount of callus was observed in the TSD‐7d, TSD‐14d, and TSD‐21d groups of mice (Figure 1B). Safranin O‐fast green staining revealed that the cartilage formation in the femoral fractures of the model group was reduced compared to the sham group. After the TSD intervention treatment for 7, 14, and 21 days, chondrogenesis gradually increased (Figure 1C). In addition, IF showed that the expressions of Emcn and CD31 were significantly lower in the model group than in the sham group. Emcn and CD31 expressions gradually increased after 7, 14, and 21 days of TSD intervention (Figure 1D, E). Runx2 and Osterix are essential for osteoblast differentiation [31]. MMP‐9 is involved in bone development, remodeling, and repair, as well as in the degradation of the extracellular matrix [32]. PDGF‐BB and SLIT3 are involved in the coupling of angiogenesis and osteogenesis [14]. Our findings indicated that the levels of osteogenic markers, including Osterix, Runx2, and MMP‐9, as well as angiogenic markers PDGF‐BB and SLIT3, were suppressed in the model group compared to the sham group. However, these levels gradually elevated after 7, 14, and 21 days of TSD intervention (Figure 1F). These results suggested that TSD affected H‐type angiogenesis during the healing process of fractured mice.

Figure 1TSD affected H‐type angiogenesis during the healing of fractured mice. (A) Micro‐CT was utilized to scan the fracture end of the mouse and establish a three‐dimensional map, and the BV/TV (%) and BMD (g/cm^3^)of the fracture site of the mouse femur were determined. (B) H&E staining of morphological lesions of the femur in mice. (C) Safranin O‐fast green staining of the distribution of femur bone in mice. (D, E) IF staining of Emcn and CD31 expressions in the mouse femur. Scale bar = 25 μm, magnification = 400×. (F) qRT‐PCR detection of osteogenic indexes Osterix, Runx2, and MMP‐9, and angiogenic indexes PDGF‐BB and SLIT3 levels in mouse femur. For animal experiments, *n* = 5;  ^∗^ *p* < 0.05 vs. sham, ^#^ *p* < 0.05 vs. model.

**Figure:**
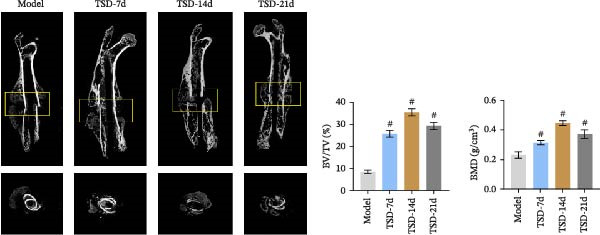
(A)

**Figure:**
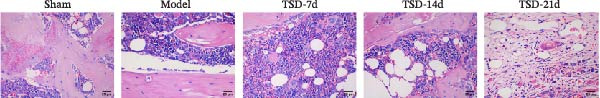
(B)

**Figure:**
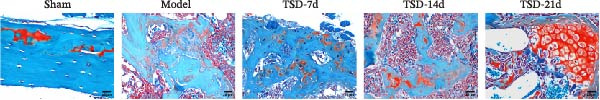
(C)

**Figure:**
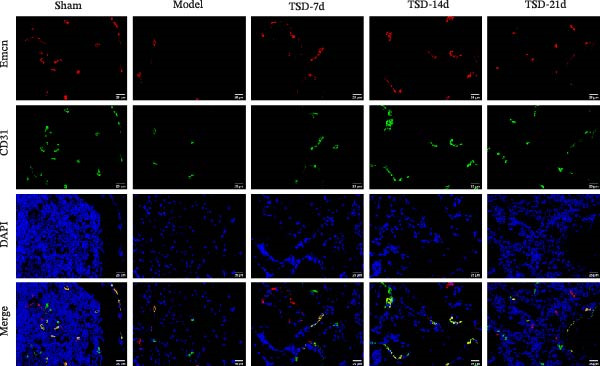
(D)

**Figure:**
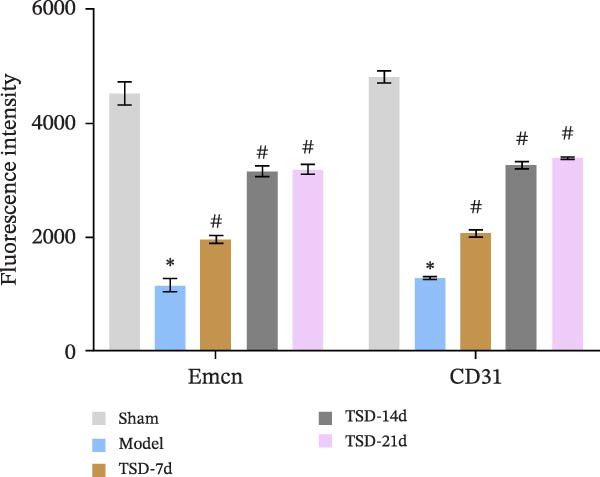
(E)

**Figure:**
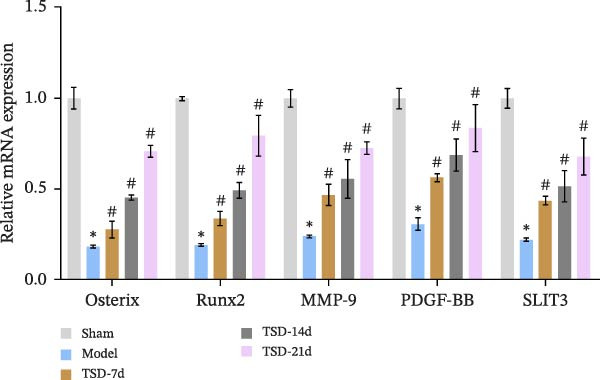
(F)

### 3.2. TSD Affected the Homing of MSCs During the Healing of Fractured Mice

**Figure 2:**
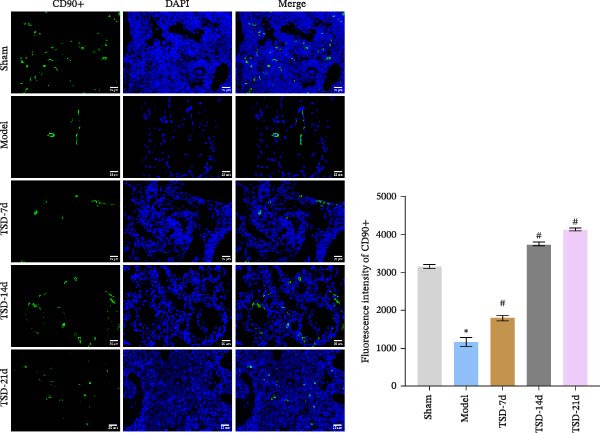
TSD affected the homing of MSCs during the healing of fractured mice. IF staining of the MSCs marker CD90. Scale bar = 25 μm, magnification = 400×. For animal experiments, *n* = 5;  ^∗^ *p* < 0.05 vs. sham, ^#^ *p* < 0.05 vs. model.

MSCs express the specific marker CD90 [33]. IF demonstrated that CD90 expression was lower in the model group, compared to the sham group. However, after 7, 14, and 21 days of TSD intervention, CD90 expression gradually increased (Figure 2). These results indicated that TSD influenced the homing of MSCs during the healing process in fractured mice.

### 3.3. TSD Affected pVHL/HIF‐1α/VEGF Axis During the Healing of Fractured Mice

We evaluated the levels of pVHL, HIF‐1α, and VEGF, and nuclear expression of HIF‐1α. Compared to the sham group, the model group exhibited decreased levels of HIF‐1α and VEGF, along with a reduced nuclear expression of HIF‐1α. In contrast, the expression of pVHL was increased in the model group. However, after 7, 14, and 21 days of TSD treatment, we observed a gradual increase in HIF‐1α and VEGF levels, as well as nuclear expression of HIF‐1α, while the expression of pVHL gradually decreased (Figure 3A, B). Our results suggested that TSD affected the pVHL/HIF‐1α/VEGF axis during the healing of fractured mice.

Figure 3TSD affected the pVHL/HIF‐1α/VEGF axis during the healing of fractured mice. (A, B) Western blot analysis of pVHL, HIF‐1α, and VEGF levels, and nuclear expression of HIF‐1α. For animal experiments, *n* = 5;  ^∗^ *p* < 0.05 vs. sham, ^#^ *p* < 0.05 vs. model.

**Figure:**
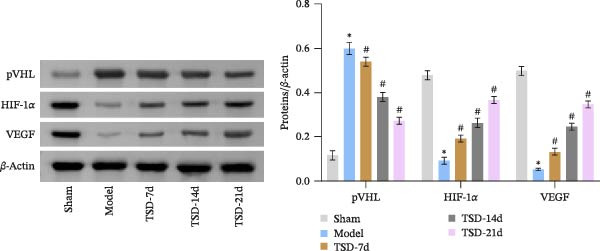
(A)

**Figure:**
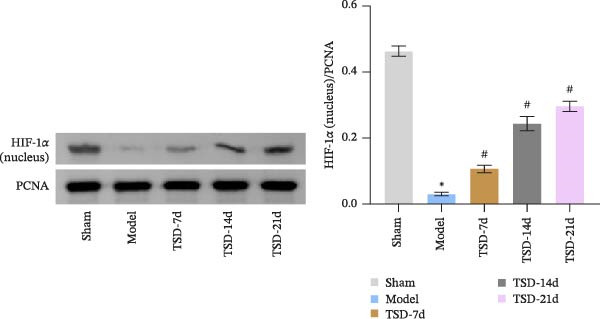
(B)

### 3.4. TSD Enhanced Proliferation and Migration of MSCs and Inhibition of pVHL/HIF‐1α Ubiquitination

We further examined the function of TSD through in vitro cell experiments. We initially treated MSCs with serum containing 0%, 5%, 10%, and 20% TSD for 24 h. Our findings indicated that a concentration of 10% TSD‐containing serum significantly affected endothelial cell function while avoiding the cytotoxicity associated with higher concentrations (Figure S1A). Therefore, we proceeded to treat MSCs with control serum and 10% TSD‐containing serum for further study. MSCs in the TSD serum group exhibited increased activity and migration ability compared to those in the control serum group (Figure 4A, B). Moreover, we observed that, in comparison to the control serum group, levels of HIF‐1α and VEGF, as well as the nuclear expression of HIF‐1α and its ubiquitination levels, were decreased in the TSD serum group, while pVHL expression showed a reduction (Figure 4C, D). Co‐IP was utilized to detect the interaction between pVHL and HIF‐1α in the model and TSD groups. We found that the interaction between pVHL and HIF‐1α was diminished in the TSD group compared to the model group. This suggests that TSD promotes the stabilization of HIF‐1α by disrupting the pVHL‐mediated degradation pathway (Figure 4E). Furthermore, we overexpressed VHL in MSCs, resulting in an elevated pVHL level in the oe‐VHL group compared to the oe‐NC group (Figure 4F). HIF‐1α levels in the oe‐VHL group decreased compared to the oe‐NC group. We also administered MG132 treatment, which increased HIF‐1α levels (Figure 4G). Our results revealed that TSD enhanced the proliferation and migration of MSCs, while also inhibiting the ubiquitination of pVHL/HIF‐1α.

Figure 4TSD enhanced proliferation and migration of MSCs and inhibition of pVHL/HIF‐1α ubiquitination. (A) CCK‐8 assay evaluation of cell viability. (B) Transwell assay measurement of cell migration. Scale bar = 100 μm, magnification = 100×. (C) Western blot analysis of pVHL, HIF‐1α, and VEGF levels, and nuclear expression of HIF‐1α.  ^∗^ *p* < 0.05 vs. control serum. (D) Detection of HIF‐1α ubiquitination level. (E) Co‐IP detection of direct binding of pVHL and HIF‐1α. (F) Western blot detection of pVHL level.  ^∗^ *p* < 0.05 vs. oe‐NC, ^#^ *p* < 0.05 vs. TSD. (G) Western blot detection of HIF‐1α level.  ^∗^ *p* < 0.05 vs. oe‐NC, ^#^ *p* < 0.05 vs. oe‐VHL. For cell experiments, *n* = 3.

**Figure:**
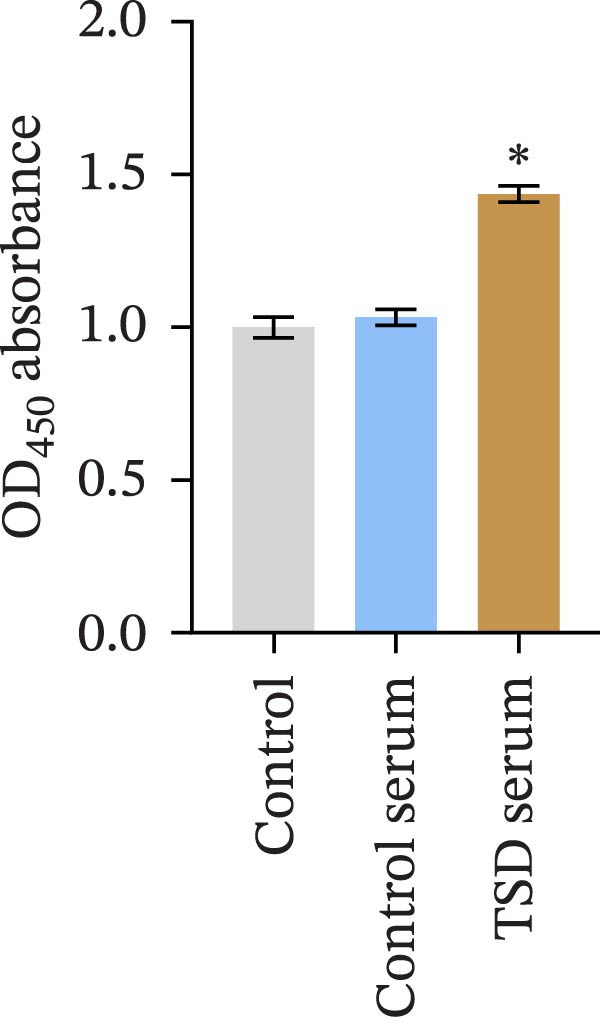
(A)

**Figure:**
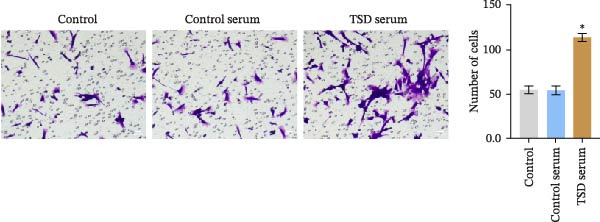
(B)

**Figure:**
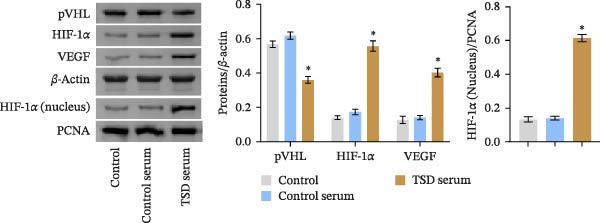
(C)

**Figure:**
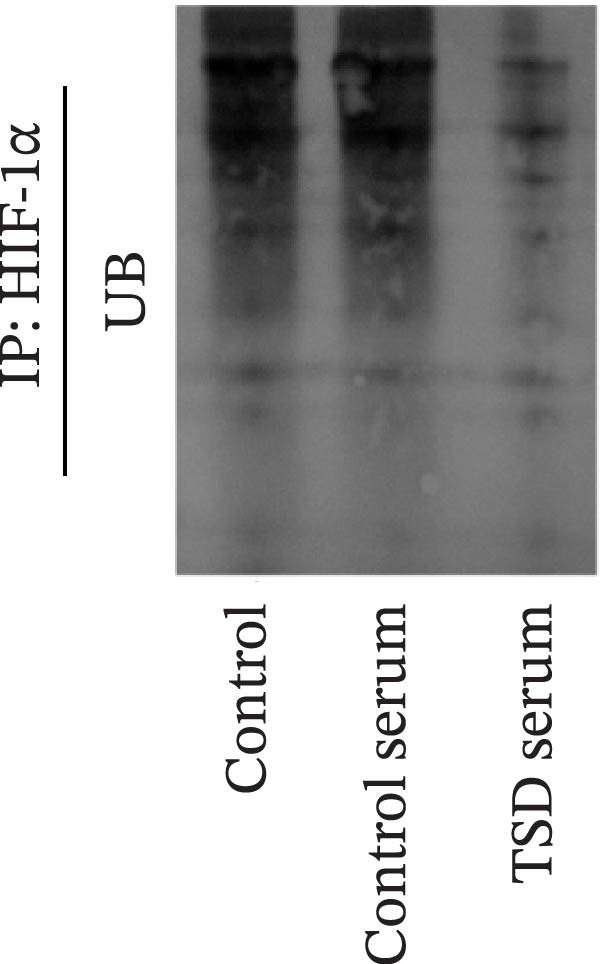
(D)

**Figure:**
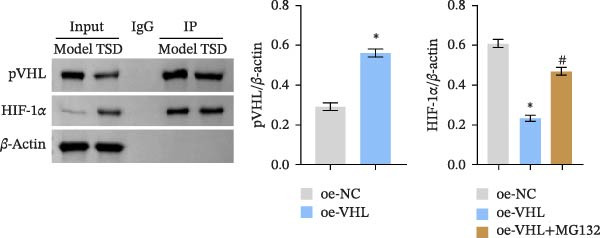
(E)

**Figure:**
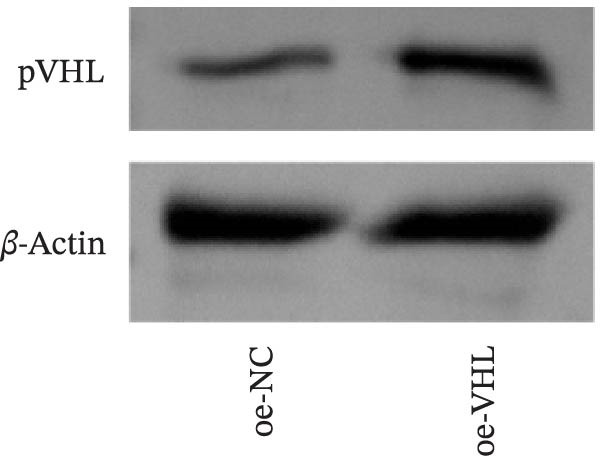
(F)

**Figure:**
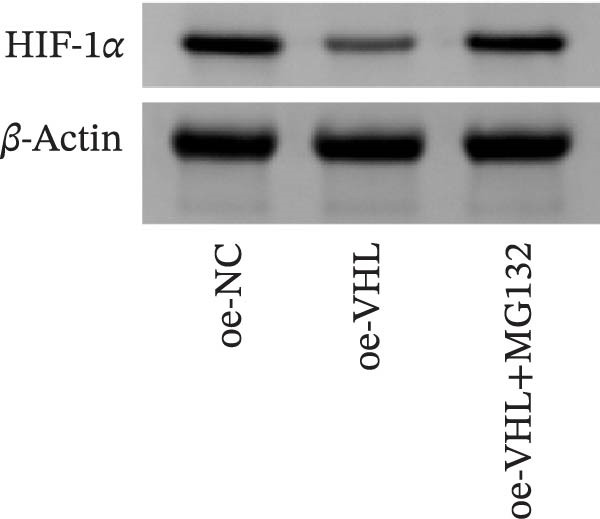
(G)

### 3.5. Molecular Docking Verification of the Binding of Effective Compounds in TSD and pVHL

According to our previous research, five compounds were detected in TSD: ferulic acid, 5‐hydroxymethylfurfural, amygdalin, hydroxysafflor yellow A, and paeoniflorin [20]. We further examined the binding of these compounds to pVHL using molecular docking. We discovered that the binding energy of ferulic acid to pVHL is −7.0 kcal/mol, which is less than −5 kcal/mol, indicating that this compound can bind to protein spontaneously. The interactions primarily occur through hydrogen bonds and hydrophobic interactions. Ferulic acid can form stable hydrogen bonds with ARG 86, LYS 162, and TYR 157 in the protein. Additionally, its hydrophobic groups can interact with PRO 158, which are the main forces promoting its binding to the active site (Figure 5A). For hydroxysafflor yellow A, the binding energy to pVHL is −6.4 kcal/mol, also indicating spontaneous binding. This compound forms a stable hydrogen bond with ARG 231 of the Gnpat protein at a distance of 2.86 Å. Furthermore, its hydrophobic groups can interact with amino acids HIS 147, GLY 229, ALA 171, and GLY 173 in the protein A chain, facilitating its binding to the active site (Figure 5B). The binding energy of 5‐hydroxymethylfurfural docking with pVHL is −4.4 kcal/mol, which is greater than −5 kcal/mol, indicating weak binding to pVHL (Figure 5C). The binding energy of amygdalin to pVHL is −7.9 kcal/mol, indicating that it can spontaneously bind to the protein. This compound interacts mainly through hydrogen bonds and hydrophobic interactions, forming stable hydrogen bonds with THR 123, GLU 126, ARG 133, ASP 156, LYS 162, ASP 163, and PRO 158. Its hydrophobic groups can interact with LEU 95 and PRO 120, which contribute to the compound’s binding to the active site (Figure 5D). The binding energy of paeoniflorin to pVHL is −8.1 kcal/mol, indicating that it can also spontaneously bind to the protein. This compound primarily interacts through hydrogen bonds and hydrophobic interactions, forming stable hydrogen bonds with LYS 162, GLY 93, GLU 126, ASP 156, and ASP 163. Its hydrophobic groups can interact with LYS 162, further promoting its binding to the active site (Figure 5E). To validate the effectiveness of the experimental methods and ensure the reliability of the results, we selected 5‐hydroxymethylfurfural with the lowest molecular docking score with pVHL and a binding energy greater than −5 kcal/mol for the DARTS assay as a supplementary experiment. Cells were treated with various concentrations of 5‐hydroxymethylfurfural (0, 20, 40, 60, and 80 μM) in combination with pronase. Subsequently, the expression of pVHL was detected using western blot. The experimental data revealed that compared with the control group, the expression of pVHL in the pronase + 5‐hydroxymethylfurfural groups was significantly reduced. However, within the tested concentration range, 5‐hydroxymethylfurfural did not significantly influence the stability of pVHL (Figure S1B). This finding provided an effective negative control for our molecular docking experiments. In addition, HPLC was utilized to detect the compounds with the highest binding energy to pVHL in the peripheral blood of TSD group, which includes ferulic acid, amygdalin, hydroxysafflor yellow A, and paeoniflorin (Figure 5F).

Figure 5Molecular docking verification of the binding of effective compounds in TSD and pVHL. (A–E) Molecular docking of the compounds ferulic acid, hydroxysafflor yellow A, 5‐hydroxymethylfurfural, amygdalin, and paeoniflorin with pVHL. (F) HPLC of the compounds with the highest binding energy to pVHL in the peripheral blood of the TSD group.

**Figure:**
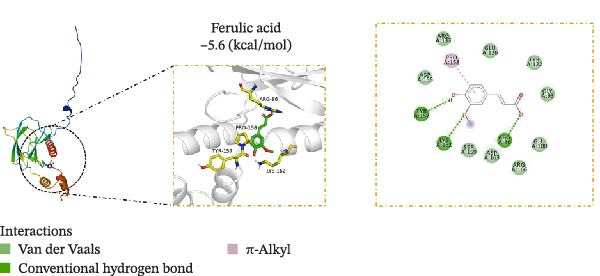
(A)

**Figure:**
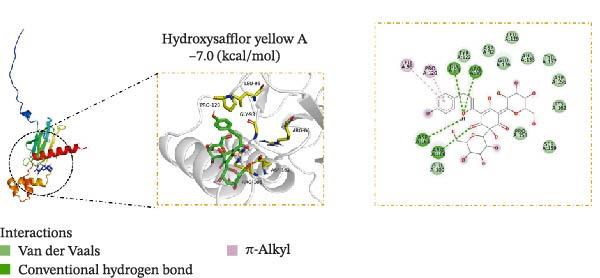
(B)

**Figure:**
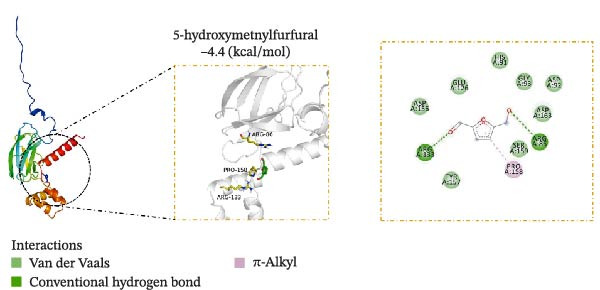
(C)

**Figure:**
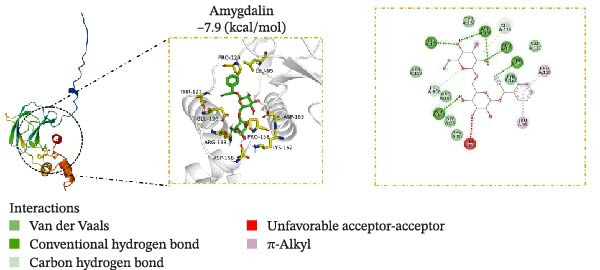
(D)

**Figure:**
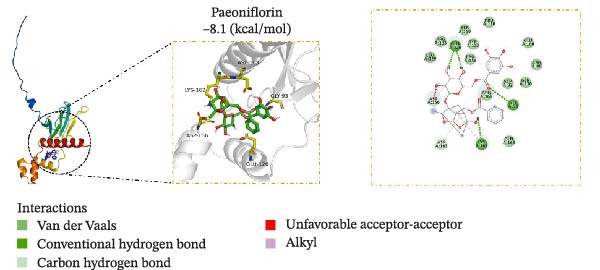
(E)

**Figure:**
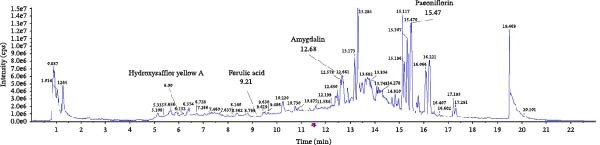
(F)

### 3.6. Paeoniflorin Promoted the Proliferation and Migration of MSCs Through pVHL/HIF‐1α Pathway to Promote Angiogenesis

We treated MSCs with various concentrations of paeoniflorin (20, 40, 60, 80, 100, and 120 μM) to assess its effects on cell viability. As the concentration of paeoniflorin increased, the viability of MSCs gradually increased. However, at higher concentrations of 100 and 120 μM, the viability of the MSCs significantly decreased. Notably, MSCs treated with 80 μM paeoniflorin exhibited the highest viability (Figure 6A). Furthermore, we measured the protein levels of pVHL in the MSCs. As the paeoniflorin concentration increased from 20 to 80 μM, the levels of pVHL protein gradually decreased. Conversely, at concentrations of 100 and 120 μM, pVHL protein levels increased. The lowest levels of pVHL protein were observed in the MSCs treated with 80 μM paeoniflorin (Figure 6B). Therefore, we selected 80 μM paeoniflorin for further study. The DARTS assay confirmed the binding of paeoniflorin to pVHL. In the presence of pronase, as the concentration of paeoniflorin treatment increased, the protein levels of pVHL gradually increased (Figure 6C). We also used the HIF‐1α inhibitor 2‐ME2. Compared to the DMSO group, the viability and migration ability of MSCs in the 80 μM paeoniflorin group increased. However, after treatment with 2‐ME2, both the viability and migration ability of the MSCs decreased (Figure 6D, E). Moreover, compared to the DMSO group, the expression of HIF‐1α and VEGF, as well as the nuclear expression of HIF‐1α, increased in the 80 μM paeoniflorin group. After treatment with 2‐ME2, the expression of HIF‐1α and VEGF, as well as the nuclear expression of HIF‐1α, decreased (Figure 6F). Additionally, we extracted mouse aortic endothelial cells. The expression of CD31 on the surface of these endothelial cells was evaluated using flow cytometry and IF, revealing a positive rate of 97.91%, indicating successful extraction (Figure 6G, H). Furthermore, MSCs in the control, DMSO, 80 μM paeoniflorin, and 80 μM paeoniflorin + 2‐ME2 groups were cocultured with endothelial cells. Cell viability in the 80 μM paeoniflorin group increased compared to the DMSO group. After treatment with 2‐ME2, cell viability decreased (Figure 6I). In comparison to the DMSO group, the 80 μM paeoniflorin group exhibited an increased number of nodes, number of meshes, and total length. Following 2‐ME2 treatment, the number of nodes, meshes, and total length was reduced (Figure 6J, K). These results suggested that paeoniflorin promoted the proliferation and migration of MSCs through the pVHL/HIF‐1α pathway, thereby enhancing angiogenesis. To elucidate the specific mechanisms of paeoniflorin on the VHL/HIF‐1α signaling pathway, MSCs were assigned into five groups: control, DMSO, paeoniflorin, paeoniflorin+oe‐NC, and paeoniflorin+oe‐VHL. Cell viability of MSCs in different treatment groups was assessed using the CCK‐8 assay. Results showed that there was no significant difference in cell viability between the control and DMSO groups, indicating that DMSO treatment had no significant impact on cell viability. Compared with the DMSO group, cell viability was significantly enhanced in the paeoniflorin group. However, overexpression of VHL led to a decrease in cell viability (Figure S1C). The migratory capacity of MSCs in different treatment groups was evaluated using the Transwell assay. Compared with the DMSO group, cell migration was significantly increased in the paeoniflorin group. However, overexpression of VHL resulted in a reduction in cell migration (Figure S1D). Western blot analysis was conducted to detect the expression of pVHL, HIF‐1α, and VEGF, as well as the nuclear expression of HIF‐1α. Results revealed that compared with the DMSO group, the expression of pVHL was significantly decreased in the paeoniflorin group, while the expression of HIF‐1α and VEGF and the nuclear expression of HIF‐1α were significantly increased. Overexpression of VHL led to an increase in pVHL expression and a decrease in the expression of HIF‐1α, VEGF, and the nuclear expression of HIF‐1α (Figure S1E). These findings suggested that paeoniflorin could significantly upregulate the expression of HIF‐1α and VEGF, as well as the nuclear expression of HIF‐1α, while overexpression of VHL weakened these effects.

Figure 6Paeoniflorin promoted the proliferation and migration of MSCs through the pVHL/HIF‐1α pathway to promote angiogenesis. (A) CCK‐8 assay evaluation of cell viability. (B) Western blot analysis of pVHL levels.  ^∗^ *P* < 0.05 vs. DMSO. (C) DARTS verification of the combination of paeoniflorin with pVHL.  ^∗^ *p* < 0.05 vs. control, ^#^ *p* < 0.05 vs. pronase. (D) CCK‐8 assay evaluation of cell viability. (E) Transwell assay measurement of cell migration. Scale bar = 100 μm, magnification = 100×. (F) Western blot analysis of HIF‐1α and VEGF levels, and nuclear expression of HIF‐1α.  ^∗^ *p* < 0.05 vs. DMSO, ^#^ *p* < 0.05 vs. 80 μM paeoniflorin. (G, H) Flow cytometry and IF analysis of CD31 on the surface of endothelial cells. (I) CCK‐8 assay evaluation of cell viability. (J) Tube formation assay determination of the vascular ability of cells. Scale bar = 100 μm, magnification = 100×. (K) Number of nodes, number of meshes, and total length.  ^∗^ *p* < 0.05 vs. DMSO, ^#^ *p* < 0.05 vs. 80 μM paeoniflorin. For cell experiments, *n* = 3.

**Figure:**
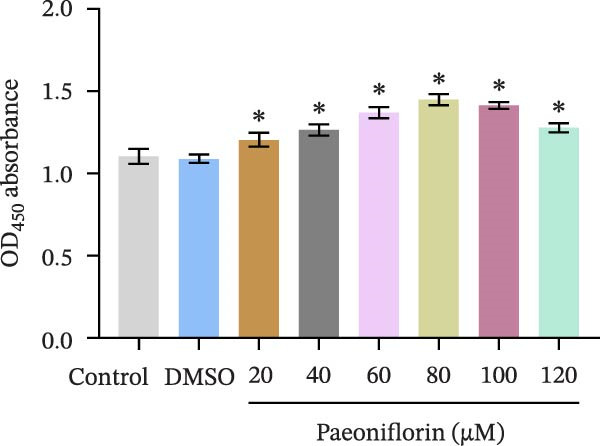
(A)

**Figure:**
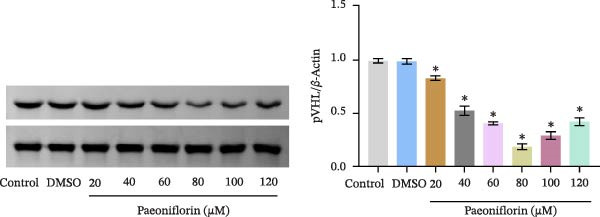
(B)

**Figure:**
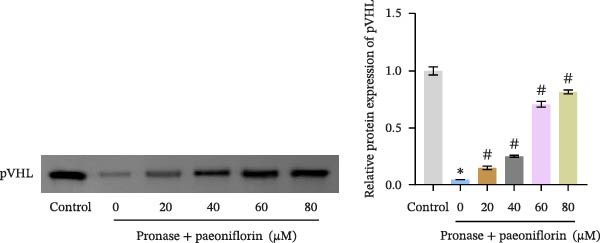
(C)

**Figure:**
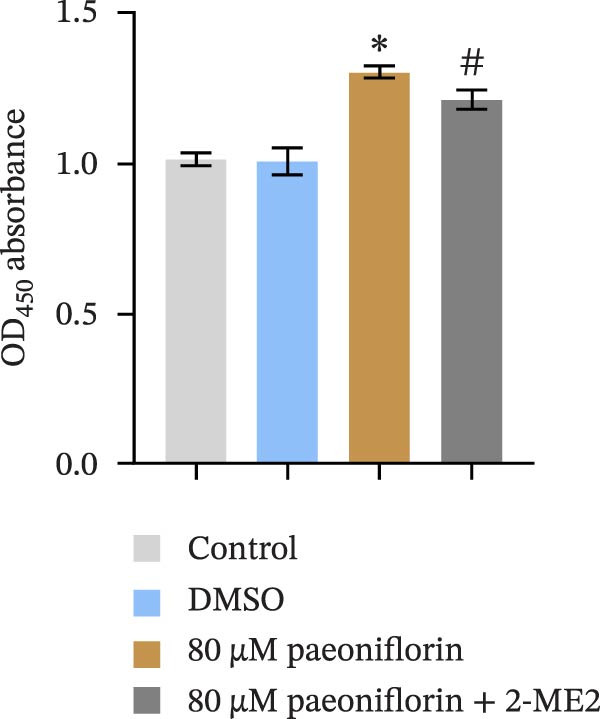
(D)

**Figure:**
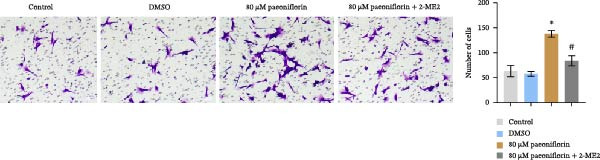
(E)

**Figure:**
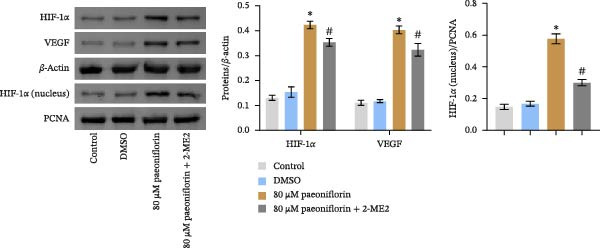
(F)

**Figure:**
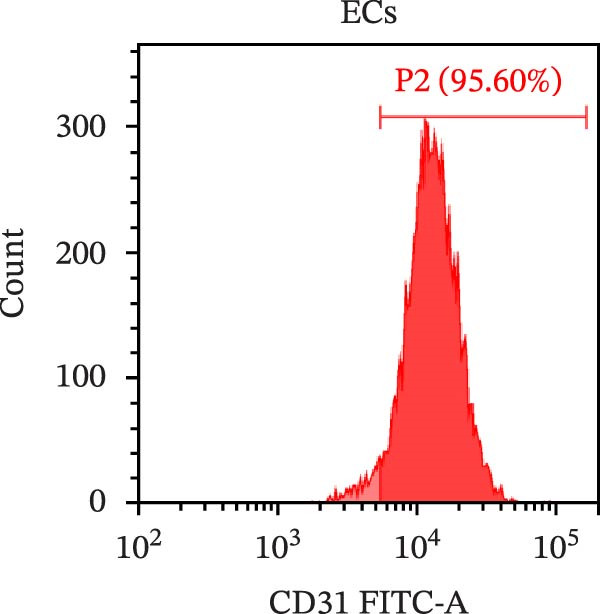
(G)

**Figure:**
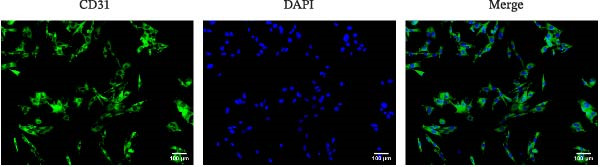
(H)

**Figure:**
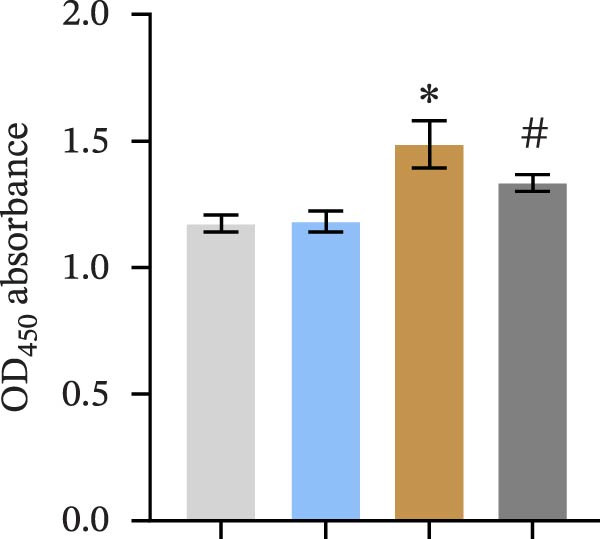
(I)

**Figure:**
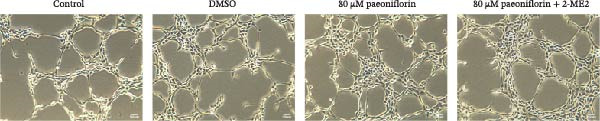
(J)

**Figure:**
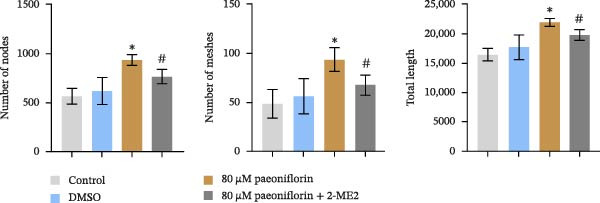
(K)

Furthermore, MSCs in the control, DMSO, paeoniflorin, paeoniflorin+oe‐NC, and paeoniflorin+oe‐VHL groups were cocultured with endothelial cells. The results showed that compared with the DMSO group, cell viability was significantly increased in the paeoniflorin group. However, overexpression of VHL led to a decrease in cell viability (Figure S1F). Additionally, compared with the DMSO group, the paeoniflorin group exhibited an increased number of nodes, meshes, and overall length. However, overexpression of VHL resulted in decreased counts of nodes, meshes, and total length (Figure S1G, H). This indicated that under the condition of coculture with endothelial cells, paeoniflorin could significantly promote tube formation and angiogenic capacity, while overexpression of VHL diminished these effects. These supplementary experimental results demonstrated that paeoniflorin could significantly enhance the viability and migratory capacity of MSCs and promote angiogenesis by upregulating the expression of HIF‐1α and VEGF. Moreover, overexpression of VHL weakened these effects, suggesting that paeoniflorin may exert its effects by modulating the VHL/HIF‐1α signaling pathway.

### 3.7. TSD Promoted Endothelial Angiogenesis in MSCs

Additionally, MSCs were cocultured with endothelial cells for 24 h. MSCs were treated with control serum, 10% TSD‐containing serum, and 10% TSD‐containing serum combined with 10 μM 2‐ME2. The findings indicated that cell viability was significantly higher in the TSD serum group compared to the control serum group. However, following treatment with 2‐ME2, cell viability decreased (Figure 7A). In comparison to the control serum group, the TSD serum group demonstrated an increased number of nodes, meshes, and overall length. After the administration of 2‐ME2, the counts of nodes, meshes, and total length all decreased (Figure 7B, C). These results suggested that TSD promoted endothelial angiogenesis in MSCs.

Figure 7TSD promoted endothelial angiogenesis in MSCs. (A) CCK‐8 assay evaluation of cell viability. (B) Tube formation assay determination of the vascular ability of cells. Scale bar = 100 μm, magnification = 100×. (C) Number of nodes, number of meshes, and total length.  ^∗^ *p* < 0.05 vs. control serum, ^#^ *p* < 0.05 vs. TSD serum. For cell experiments, *n* = 3.

**Figure:**
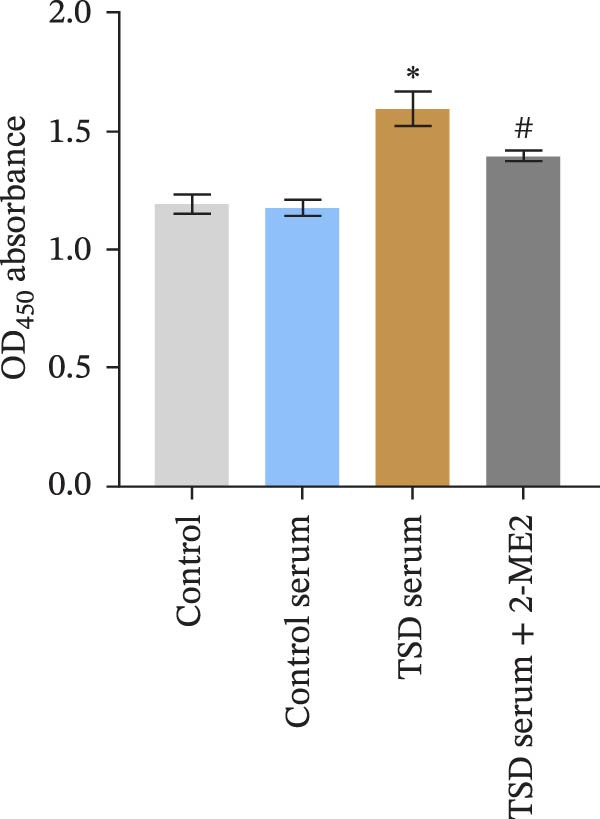
(A)

**Figure:**
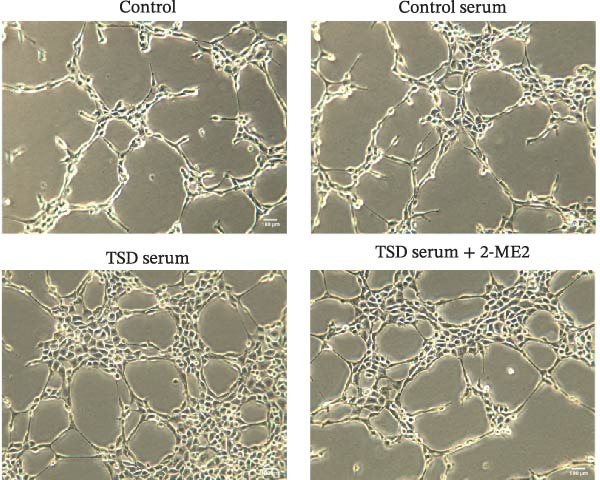
(B)

**Figure:**
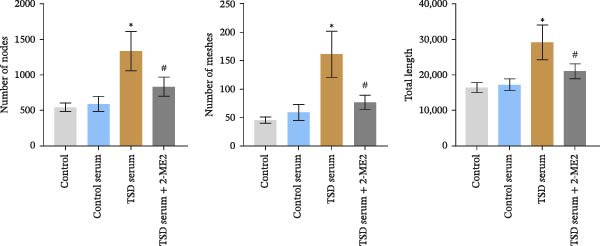
(C)

### 3.8. TSD Affected H‐Type Angiogenesis and MSCs Homing During the Healing Process of Fractured Mice Through HIF‐1α Axis

Finally, we administered 2‐ME2 to fractured mice for 21 days. H&E staining revealed that fibrous and cartilaginous calluses were present at the fracture site in both the model and 2‐ME2 groups. Notably, a significant amount of callus formation was observed in the TSD group. However, the TSD + 2‐ME2 group showed a reversal of the effects seen with TSD treatment (Figure 8A). Safranin O‐fast green staining demonstrated that, compared to the model group, cartilage formation at the femoral fracture site was enhanced in the TSD group, while it was reduced in the 2‐ME2 group. Additionally, when comparing the TSD group to the TSD + 2‐ME2 group, cartilage formation in the latter was also diminished (Figure 8B). IF analysis showed an increase in the expressions of CD90, Emcn, and CD31 in the TSD group compared to the model group. Conversely, these expressions were lower in the 2‐ME2 group (Figure 8C–E). Furthermore, the osteogenic markers Osterix, Runx2, and MMP‐9, as well as the angiogenic markers PDGF‐BB and SLIT3, were elevated in the TSD group compared to the model group. In contrast, these markers were decreased in the 2‐ME2 group (Figure 8F). However, the TSD + 2‐ME2 group reversed the changes of the above indexes treated by TSD (Figure 8C–F). These results suggested that TSD affected H‐type angiogenesis and MSCs homing during the healing process of fractured mice via the HIF‐1α axis.

Figure 8TSD affected H‐type angiogenesis and MSCs homing during the healing process of fractured mice through the HIF‐1α axis. (A) H&E staining of morphological lesions of the femur in mice. (B) Safranin O‐fast green staining of the distribution of femur bone in mice. (C) IF staining of the MSCs marker CD90. (D) IF staining of Emcn and CD31 expressions in mouse femur. Scale bar = 25 μm, magnification = 400×. (E) Statistical analysis of subpart (D). (F) qRT‐PCR analysis of osteogenic indexes Osterix, Runx2, and MMP‐9, and angiogenic indexes PDGF‐BB and SLIT3 levels in mouse femur. For animal experiments, *n* = 5;  ^∗^ *P* < 0.05 vs. model, ^#^ *p* < 0.05 vs. TSD, ^&^ *p* < 0.05 vs. 2‐ME2.

**Figure:**
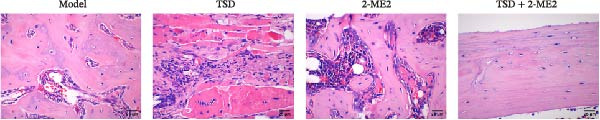
(A)

**Figure:**
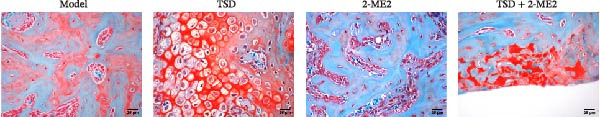
(B)

**Figure:**
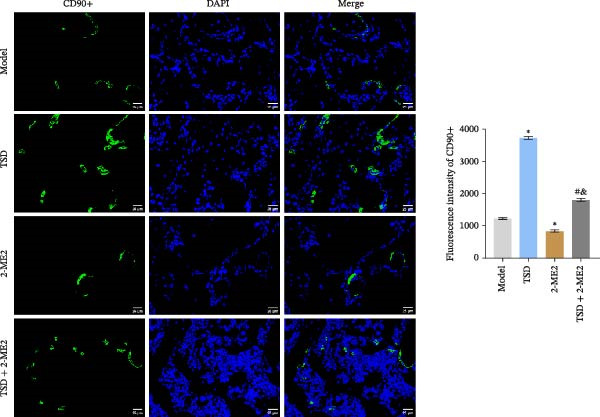
(C)

**Figure:**
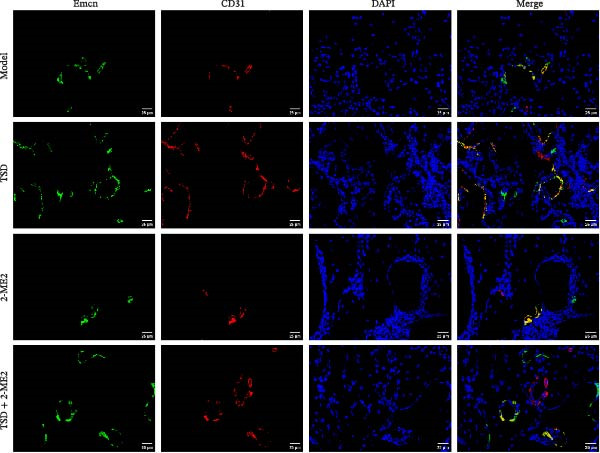
(D)

**Figure:**
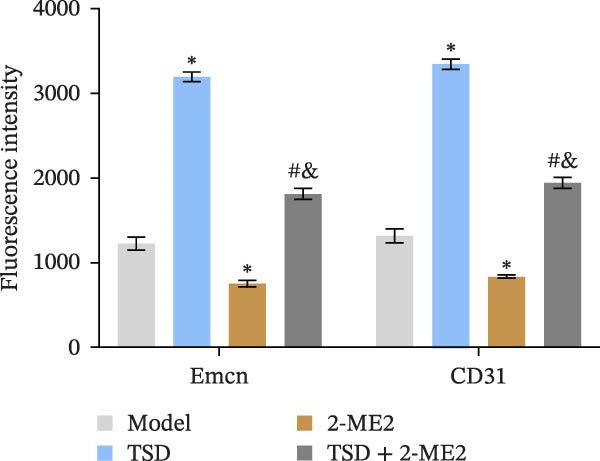
(E)

**Figure:**
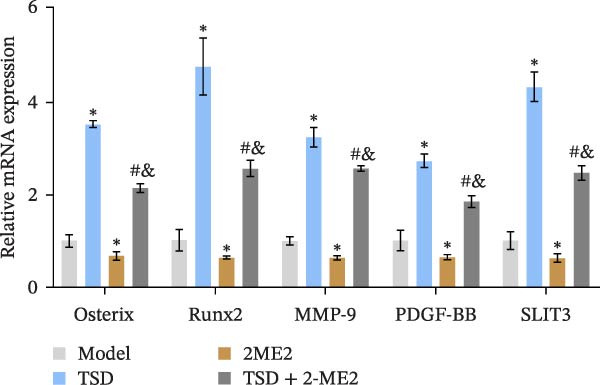
(F)

## 4. Discussion

Fracture healing is a highly intricate physiological process that requires effective communication between immune cells and stromal cells to coordinate the repair and regeneration of damaged tissue [34]. Despite advancements in orthopedics, there is still a need for treatments that can expedite fracture healing [35]. In this research, we investigated the mechanisms by which TSD promotes fracture healing through in vivo and in vitro experiments. Our findings demonstrate that TSD regulates MSC‐mediated H‐type angiogenesis by influencing VHL/HIF‐1α ubiquitination, thereby accelerating the healing process. This research is the first to reveal how TSD enhances fracture healing via the VHL/HIF‐1α ubiquitination pathway. This pathway had not been previously explored in the context of TSD and fracture healing, thus providing a novel theoretical framework for understanding the therapeutic potential of TSD.

Blood vessels are a multifunctional transport system that plays a vital role in organ development, regeneration, and the behavior of stem cells [36]. A special subtype of capillaries, referred to as H‐type vessels, exhibit unique functional characteristics during angiogenesis and osteogenesis [37]. H‐type vessels develop in the cancellous bone region during the development of long bones, providing adequate nutritional support to the cells near the growth plate. Furthermore, these vessels influence the proliferation and differentiation of bone progenitor cells and osteoclasts through paracrine signaling, thereby creating an optimal microenvironment that enhances new bone formation [38]. Therefore, targeting H‐type angiogenesis may provide a novel approach for treating various bone diseases [39]. In this research, we established a mouse fracture model and performed TSD intervention. Our results demonstrated that TSD affects H‐type angiogenesis during the healing process in fractured mice. We have confirmed that TSD can influence the formation of H‐type blood vessels, which possess unique functional characteristics and play a crucial role in angiogenesis and osteogenesis [37]. This finding expands the current understanding of how traditional Chinese medicine regulates specific vascular subtypes to promote fracture healing. Consequently, we intend to further explore the mechanisms underlying TSD’s effects.

Cell recruitment, migration, and homing to the fracture site are critical for initiating inflammatory processes, promoting angiogenesis, chondrogenesis, osteogenesis, and ultimately facilitating bone remodeling [40]. Stem cell therapy and cytokines have the potential to enhance bone repair by promoting the migration and homing of stem cells to the site of injury [41]. Bone marrow serves as a vital source of stem cells that contribute to fracture healing [42]. Studies have shown that different stages of fracture healing affect the homing of culture‐expanded MSCs [43]. By implanting a greater number of exogenous MSCs into the fracture site, overall fracture healing and bone strength can significantly improve [44]. Among these processes, the homing of bone marrow MSCs is the first step of bone formation. MSCs must migrate to the bone surface and subsequently differentiate into osteoblasts to enhance bone repair [45]. In this study, we further established that TSD influences the homing and spatial interaction of MSCs with H‐type vessels during the healing of fractured mice.

HIF‐1α is a major regulator of the body’s response to hypoxia and is essential for bone formation, remodeling, and homeostasis [46]. Additionally, HIF‐1α acts as a vital positive regulator of H‐type angiogenesis [47]. By activating HIF‐1α and osteogenesis‐related genes, it can significantly improve fracture healing, increase BMD, and increase bone strength [48]. pVHL is a tumor suppressor, and it is primarily recognized for its role in regulating HIF activity [49]. Under normal physiological conditions, pVHL promotes the ubiquitination and subsequent degradation of HIF‐1α [50]. Studies have shown that a deficiency in pVHL can markedly promote angiogenesis by elevating HIF‐1α expression [51]. A study by Hamushan et al. [52] demonstrated that high‐purity magnesium pins facilitate bone consolidation in distraction osteogenesis models by activating the VHL/HIF‐1α/VEGF pathway. Furthermore, Tang et al. [53] reported that safflower yellow promotes angiogenesis via regulating the pVHL/HIF‐1α/VEGF pathway, thus promoting osteogenic differentiation. Their research also indicated that TSD fosters fracture‐related osteogenic angiogenesis through the same pathway [54]. In this study, we further demonstrated in vivo that TSD affects the HIF‐1α axis during the healing of fractured mice. Specifically, MSCs migrate to the injury site, release angiogenic factors such as VEGF, and stimulate angiogenesis in endothelial cells. This mechanism is crucial for tissue repair and regeneration. Gaining a deeper understanding of this process provides a theoretical foundation for developing new therapeutic strategies, particularly in promoting wound healing, improving treatments for ischemic diseases, and advancing the field of tissue engineering. Consequently, we aim to further investigate the specific mechanisms involved in TSD and the pVHL/HIF‐1α/VEGF pathway during fracture healing.

Ubiquitination is a pivotal posttranslational modification in which a portion of ubiquitin is covalently bound to target proteins, thereby influencing protein stability, interaction partners, and biological functions [55]. This intricate and dynamic process regulates various cellular functions, encompassing the targeting of proteins for degradation, cell cycle progression, DNA damage repair, and numerous cell signaling pathways [56]. The ubiquitin/proteasome system is essential for maintaining the stability of Runx2 and JunB, which are key proteins required for the differentiation of mesenchymal progenitor cells and stem cells into osteoblasts [57]. Jiang et al. [58] revealed that the exosome miR‐25 derived from bone marrow MSCs regulates the ubiquitination and subsequent degradation of Runx2 through SMURF1, promoting fracture healing in mice. Huang et al. [59] demonstrated that overexpression of the E3 ubiquitin ligase WWP1 or Smurf2 results in the degradation of the target protein KLF5 in BMSCs through ubiquitination, ultimately inhibiting fracture healing. Further research indicated that TSD enhances the proliferation and migration of MSCs while inhibiting the ubiquitination of pVHL/HIF‐1α. Molecular docking studies confirmed that effective compounds such as ferulic acid, amygdalin, hydroxysafflor yellow A, and paeoniflorin exhibit strong binding affinity to pVHL. The high binding capacity of these compounds to pVHL in the peripheral blood of the TSD group was detected using HPLC. Paeoniflorin, a natural compound extracted from *Paeonia lactiflora*, is known for its significant anti‐inflammatory and immunomodulatory effects [60]. Paeoniflorin has demonstrated considerable antitumor effects against various forms of tumors, such as liver cancer, gastric cancer, breast cancer, lung cancer, pancreatic cancer, colorectal cancer, and bladder cancer. It induces apoptosis and inhibits cell proliferation, invasion, and metastasis through diverse molecular mechanisms [61]. Additionally, research indicates that both tetramethylpyrazine and paeoniflorin inhibited oxidized low‐density lipoprotein‐induced angiogenesis in human umbilical vein endothelial cells via the VEGF and Notch pathways [62]. However, the efficacy of paeoniflorin in bone fracture healing remains unclear. In this study, we further investigate the role of paeoniflorin in fracture healing and the mechanisms involved. Our findings reveal that paeoniflorin enhances the proliferation and migration of MSCs through the pVHL/HIF‐1α pathway to promote angiogenesis. Specifically, paeoniflorin binds to pVHL and promotes the degradation of pVHL. pVHL promotes the ubiquitination of HIF‐1α, leading to its degradation.

5‐Hydroxymethylfurfural has been found to react with vitamin C, resulting in a decrease in intracellular vitamin C levels. This reaction can affect the activity of proline hydroxylase, which is responsible for stabilizing HIF‐1α. Since pVHL facilitates the degradation of HIF‐1α by binding to its hydroxylated sites, 5‐hydroxymethylfurfural indirectly affects the function of pVHL by reducing the vitamin C levels [63]. Amygdalin exhibits cell cycle regulatory effects associated with pVHL in certain cell lines. For example, in the A498 cell line, the combination of amygdalin and SFN (thiosulfonamide) significantly increases the proportion of cells in the G0/G1 phase while decreasing those in the G2/M phase. This phenomenon may be connected to the expression status of pVHL, as A498 cells completely lack pVHL, while other cell lines, such as Caki‐1 and KTCTL‐26, express wild‐type pVHL and carry mutations in the pVHL gene, respectively [64]. Despite the findings discussed, the direct interactions of ferulic acid, hydroxysafflor yellow A, and other compounds with pVHL remain inadequately investigated. This study lays the groundwork for future research, including comprehensive investigations into the active components in TSD, such as ferulic acid, hydroxysafflor yellow A, amygdalin, and paeoniflorin, and their specific roles in fracture healing. Such investigations could lead to the development of new drugs or therapeutic formulations incorporating these components. Furthermore, our coculture studies revealed that TSD promotes endothelial angiogenesis in MSCs. In vivo experiments also confirmed that TSD significantly affected H‐type angiogenesis and MSCs homing during the healing process in fractured mice through the HIF‐1α axis. Our study demonstrated both in vivo and in vitro that TSD regulates MSC‐mediated H‐type angiogenesis through pVHL/HIF‐1α ubiquitination, thereby accelerating fracture healing. These findings suggested the potential for combining TSD with other therapeutic methods for enhancing fracture healing, such as using exogenous MSCs or growth factors, which could be explored in future clinical trials.

However, due to the limitations of laboratory conditions, we are currently unable to conduct biomechanical tests to assess bone stiffness, strength, and toughness. Despite this constraint, we can provide detailed insights into the fracture healing process and quality through radiological assessments. The results of the micro‐CT scans indicated that the TSD intervention had a significant impact on fracture healing. These findings provide strong support for our study, demonstrating that the TSD intervention could significantly promote fracture healing. We recognize the importance of biomechanical testing in evaluating the quality of fracture healing and plan to integrate this testing method in future studies.

In conclusion, we examined the mechanism of TSD in fracture healing and identified its potential mechanism of action related to VHL/HIF‐1α ubiquitination and MSC‐mediated H‐type angiogenesis. Our results suggest a promising new approach of treating fracture healing with TSD. This study integrates traditional Chinese medicine with modern molecular biology, highlighting the potential of TSD in modulating key pathways in fracture healing. This integration provides a theoretical basis for developing innovative therapeutic strategies that combine traditional therapies with modern scientific methods. Additionally, a deeper understanding of the role of VHL/HIF‐1α ubiquitination and MSC‐mediated H‐type angiogenesis in fracture healing may offer a theoretical foundation for the development of novel treatment approaches for fracture healing.

## Author Contributions

Material preparation, experiments, data collection, and analysis were performed by Wangyang Li, Zebing Ma, Peng He, Wuji Xu, Xiaolan Liu, Jinlong Yao, Qiyao Wu, Pinglan Zou, and Tiao Li. The first draft of the manuscript was written by Wangyang Li, Pinglan Zou, and Tiao Li, and all authors commented on previous versions of the manuscript and contributed to the study conception and design.

## Funding

This work was supported by the Science and Technology Innovation Program of Hunan Province, China (Grant 2023RC3167), the Natural Science Foundation of Hunan Province, China (Grants 2024JJ6349 and 2025JJ80902), the Research Foundation of Education Bureau of Hunan Province, China (Grant 24A0261), the Hunan University of Chinese Medicine (Grants 24A0261 and Z2023YYJJ10), the Construction Project of the Inheritance Studio for Veteran Pharmaceutical Workers (Grant 2024‐06‐03‐011‐001), and the Health Research Project of Hunan Provincial Health Commission (Grant 20257674).

## Ethics Statement

All experimental procedures and animal handling were performed with the approval of the Animal Care and Use Committee of the Second Affiliated Hospital of Hunan University of Chinese Medicine (Number HNUCM21‐2310‐02), Hunan University of Chinese Medicine, in accordance with the National Institutes of Health Guide for the Care and Use of Laboratory Animals, and studies involving laboratory animals follows the ARRIVE guidelines.

## Conflicts of Interest

The authors declare no conflicts of interest.

## Supporting Information

Additional supporting information can be found online in the Supporting Information section.

## Supporting information


**Supporting Information** Figure S1: Evaluation of TSD‐containing serum effects and paeoniflorin‐induced molecular mechanisms in MSCs and cocultured endothelial cells. (A) CCK‐8 assay evaluation of cell viability after 5%, 10%, and 20% TSD‐containing serum treatment.  ^∗^ *p* < 0.05 vs. control. (B) DARTS verification of the combination of 5‐hydroxymethylfurfural with pVHL.  ^∗^ *p* < 0.05 vs. control. MSCs were divided into control, DMSO, paeoniflorin, paeoniflorin+oe‐NC, and paeoniflorin+oe‐VHL groups. (C) CCK‐8 assay evaluation of cell viability. (D) Transwell assay measurement of cell migration. Scale bar = 100 μm, magnification = 100 ×. (E) Western blot analysis of pVHL, HIF‐1α and VEGF levels, and nuclear expression of HIF‐1α.  ^∗^ *p* < 0.05 vs. DMSO, ^#^ *p* < 0.05 vs. paeoniflorin+oe‐NC. MSCs in the control, DMSO, paeoniflorin, paeoniflorin+oe‐NC, and paeoniflorin+oe‐VHL groups were cocultured with endothelial cells. (F) CCK‐8 assay evaluation of cell viability. (G) Tube formation assay determination of the vascular ability of cells. Scale bar = 100 μm, magnification = 100 ×. (H) Number of nodes, number of meshes, and total length.  ^∗^ *p* < 0.05 vs. DMSO, ^#^ *p* < 0.05 vs. paeoniflorin+oe‐NC. For cell experiments, *n* = 3.

## Data Availability

The datasets used and analyzed during the current study are available from the corresponding author upon reasonable request.
