# Supplementary figures and images for: Taohong Siwu Decoction Regulates MSC‐Mediated H‐Type Angiogenesis to Accelerate Bone Fracture Healing Through VHL/HIF‐1α Ubiquitination
Source: Mediators Inflamm. 2026 Feb 16;2026:6551954. doi: 10.1155/mi/6551954 (PMC12910178; doi:10.1155/mi/6551954)

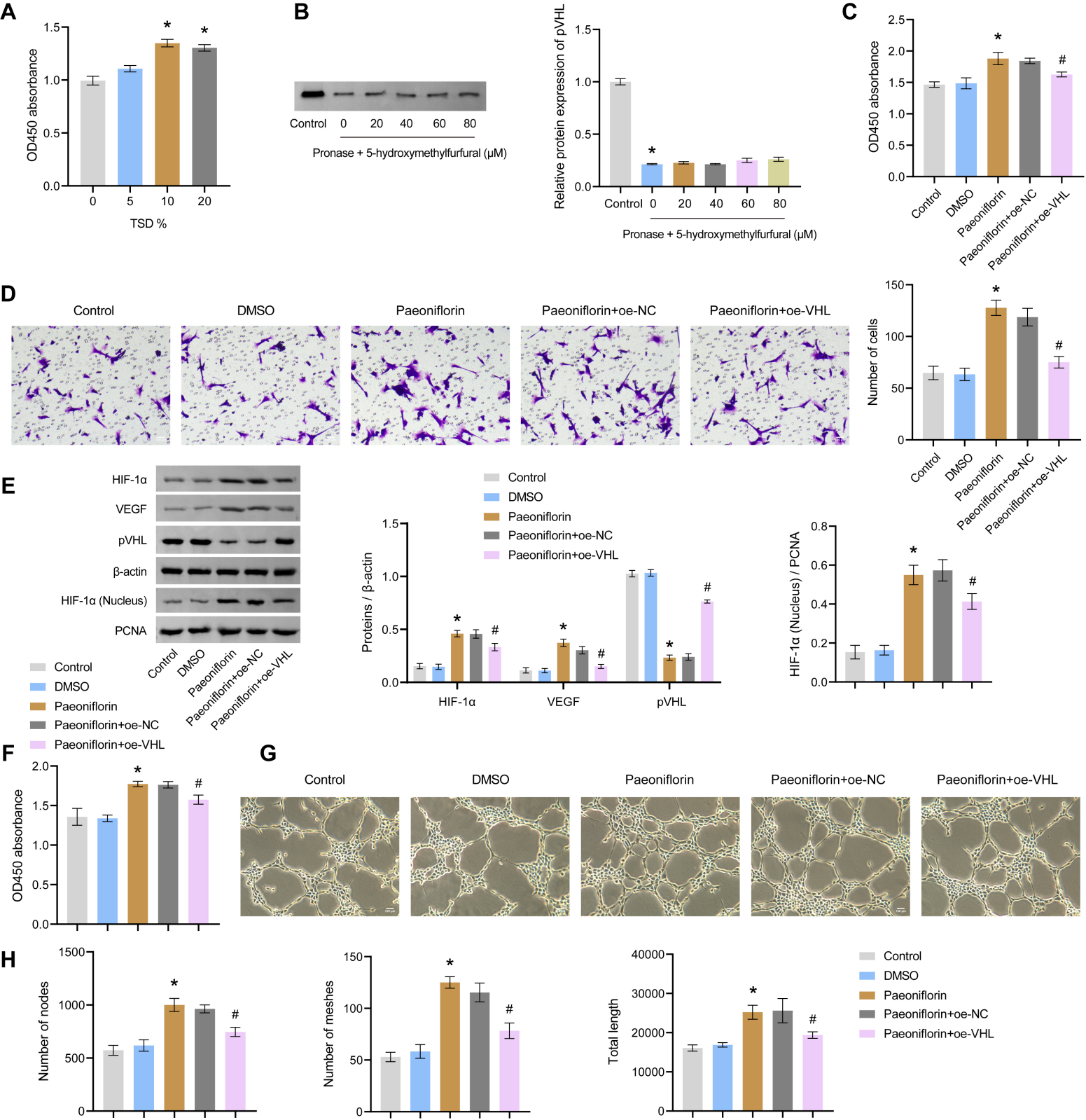

Supplement: Supplementary file 1 — Supporting Information Figure S1: Evaluation of TSD‐containing serum effects and paeoniflorin‐induced molecular mechanisms in MSCs and cocultured endothelial cells. (A) CCK‐8 assay evaluation of cell viability after 5%, 10%, and 20% TSD‐containing serum treatment. ∗ p < 0.05 vs. control. (B) DARTS verification of the combination of 5‐hydroxymethylfurfural with pVHL. ∗ p < 0.05 vs. control. MSCs were divided into control, DMSO, paeoniflorin, paeoniflorin+oe‐NC, and paeoniflorin+oe‐VHL groups. (C) CCK‐8 assay evaluation of cell viability. (D) Transwell assay measurement of cell migration. Scale bar = 100 μm, magnification = 100 ×. (E) Western blot analysis of pVHL, HIF‐1α and VEGF levels, and nuclear expression of HIF‐1α. ∗ p < 0.05 vs. DMSO, # p < 0.05 vs. paeoniflorin+oe‐NC. MSCs in the control, DMSO, paeoniflorin, paeoniflorin+oe‐NC, and paeoniflorin+oe‐VHL groups were cocultured with endothelial cells. (F) CCK‐8 assay evaluation of cell viability. (G) Tube formation assay determination of the vascular ability of cells. Scale bar = 100 μm, magnification = 100 ×. (H) Number of nodes, number of meshes, and total length. ∗ p < 0.05 vs. DMSO, # p < 0.05 vs. paeoniflorin+oe‐NC. For cell experiments, n = 3. [file MI-2026-6551954-s001.pdf]
